# Manual training of mental rotation performance: Visual representation of rotating figures is the main driver for improvements

**DOI:** 10.1177/17470218211039494

**Published:** 2021-08-13

**Authors:** Leonardo Jost, Petra Jansen

**Affiliations:** Faculty of Human Sciences, University of Regensburg, Regensburg, Germany

**Keywords:** Mental rotation, visual rotation, manual rotation, common processing

## Abstract

Studies have demonstrated that manual and mental rotation show common processes. Training studies have shown that a manual and concurrent visual rotation improves mental rotation performance. In this study, we separated the visual rotation from the manual rotation. In all, 121 participants were randomly assigned to visual training, manual rotation training, or manual training without rotational movement. Before and after the training session of 30 min, they had to solve a chronometric mental rotation test. Data were analysed with linear mixed models and showed an improvement in mental rotation performance for all groups. However, this improvement did not differ between groups. Due to the independence of the form and occurrence of the manual activity, this suggests that it is not the motor activity, but the concurrent visual rotation that leads to improvements in mental rotation tasks. Therefore, the visual component in mental rotation tasks has to be investigated in more detail.

Mental rotation is one aspect of spatial thinking. It describes the ability to imagine an object turned around in the mind (Shepard & Metzler, 1971) and can be classified as an intrinsic-dynamic spatial ability, according to a classification of Uttal et al. (2013). The process of mental rotation is extensively investigated also due to the importance for applied contexts like for mathematical performance (Mix et al., 2016). Mental rotation performance is mainly measured by two different types of tests: In a computer-based chronometric mental rotation test (Shepard & Metzler, 1971) participants are required to judge whether two rotated abstract block figures presented on the computer screen are the “same” (non-mirror reversed) or “different” (mirror reversed). The dependent variables are reaction time and error rate. Beside this, paper-pencil, psychometric mental rotation tests (Vandenberg & Kuse, 1978) exist, in which participants have to discriminate between two rotated figures and two distractors (mirrored or structurally different figures) compared to one target figure. In these tests, the number of correctly solved items are registered.

## The processing stages of mental rotation

During a mental rotation task, it has been proposed that different stages are used for processing: the perceptual stages (perceptual processing, identification and discrimination of stimuli, identification of orientation), stages of the rotation process itself (mental rotation, judgement of parity), and decision processing stages (response selection, execution) (Heil & Rolke, 2002; Just & Carpenter, 1985; Shepard & Cooper, 1986). The use of a chronometric approach allows a more detailed insight into the underlying mechanism of mental rotation. For example, reaction time at an angular disparity of 0° separates the perception and decision stage from the rotation process itself. Furthermore, a positive linear relationship between reaction time and angular disparity has been shown in chronometric mental rotation tests. This implies that participants rotate one object in the visual working memory to determine whether it matches the other object.

One point of interest is how the process of mentally rotating an object is integrated into the other stages and internally represented. The “pure insertion paradigm” proposes that an additional step of mental rotation is inserted between other processing stages of a discrimination task without interfering with other stages. This means that the mental rotation process can be added without changing the speed of other processes, such as the stimulus identification. In contrast to this, Ilan and Miller (1994) showed that participants need more time to judge if a non-rotated letter is mirror reversed or non-mirror reversed, if this task was embedded with tasks with rotated letters in comparison to only tasks with non-rotated letters. This result was not influenced by the visual quality of the stimuli and Ilan and Milller (1994) concluded that visual perception does not play an important role in the processing of mental rotation tasks, which was also confirmed in a later study of Jansen-Osmann and Heil (2007a). In two experiments, Liesefeld and Zimmer (2013) demonstrated that the representation on which the process of mental rotation works does not have a visual format.

In one classic study of Wohlschläger and Wohlschläger (1998), interference was found between mental object rotations and simultaneously executed hand movements. Considering more closely the hand movement results reported by Wohlschläger and Wohlschläger, it was shown that the performance was slower when the mental rotation was performed in the opposite direction from the hand movements. The authors suggested a common process in mental object rotation and the programming of hand movements. In a later study, Wohlschläger (2001) showed that motor planning is a crucial factor whereas preparation and execution are not as critical to performance. He concluded that mental rotation is an imagined (covert) motor action and that the interference he observed in his studies represents interference between incompatible actions. Furthermore, Gardony et al. (2014) found similar angular disparity effects in mental and physical rotations. In their study participants had to decide if two rotated objects on a screen were the ”same” (rotated) or ”different” (mirrored) either mentally or while rotating a bimanually held sensor. Moreover, their analysis demonstrated an increase of reaction time and error rate with increasing angular disparity for the “same” trials but not for “different” trials. Those studies, and also the study of Wexler et al. (1998) who demonstrated that compatible manual and mental rotation results in faster reaction times and fewer errors have triggered studies on the importance of motor processes in mental rotation and suggested a link between motor preparation and mental rotation. However, Janczyk et al. (2012) demonstrated in a series of studies that the connection between manual rotation movements and mental rotation could instead be the anticipated sensory output of the manual rotation.

Next to the above-mentioned interference studies, motor effects were also investigated in quasi-experimental designs by comparing the mental rotation performance of groups of motor experts and non-experts. A small to moderate effect of overall motor expertise (including motor activity in practice of sports and musical instruments) on mental rotation performance has been shown by Voyer and Jansen (2016). Regarding all different spatial tasks (spatial visualisation, spatial perception, and mental rotation) and specific motor expertise, studies with participants of combat sports showed the largest effects and studies with gymnasts and musicians showed medium effect sizes. In line with the proposed link between mental and manual spatial transformations, these are the sports which require the most complex spatial motor transformations.

## Training of mental rotation

First of all, it has been well established that mental rotation performance can be improved either through the repetition of the mental rotation tasks themselves or through a motor training, in which similar stimuli are rotated by participants’ movement. The investigation of specific training effects due to the repetition of mental rotation tasks of Heil et al. (1998) showed an improvement over four practice sessions. Compared to a control group, this improvement however did not transfer to untrained stimuli and untrained axes of rotation.

Similarly, Meneghetti et al. (2017) found an improvement in mental rotation performance over five training sessions both with and without the instruction of a specific rotation strategy. Contrary to the results of Heil et al., these improvements transferred to other stimuli and partially also to a psychometric mental rotation test. These differences might be due to a number of factors, including the specific practice and test trials as well as the frequency and spacing of practice sessions (Wright et al., 2008). By analysing the effects of time spent on the tasks, Jost and Jansen (2020) also demonstrated the improvement due to the repetition during a single 30-min session.

Regarding the training effects of a rotational motor training, Wiedenbauer et al. (2007) developed a manual rotation training in which participants had to rotate a joystick, which was hidden in a box. Using the joystick one of the two cube figures presented on a screen should be rotated into congruence with the other figure. The training included 192 trials, and the control group played a non-spatial computer game. Before and after the training a chronometric mental rotation test had to be completed. The manual rotation training did indeed improve mental rotation performance but specifically for those objects, which were used for training. Adams et al. (2014) replicated these results in a similar experiment. They also showed that this improvement by the manual rotation training was comparable to an improvement by repeated mental rotation tasks, further indicating that manual rotation contains similar aspects as mental rotation. The manual rotation performance, however, only improved through a manual training but not a mental training. This suggests that there are parts of the manual rotation process, which are not trained by mental rotation. The effect of manual training on mental rotation was also observed in children (Wiedenbauer & Jansen-Osmann, 2008). However, those studies do not provide any evidence that the beneficial training effect is specifically due to the motor component because the mental rotation was also visualised. In addition, it is possible that the participants rotated the stimuli mentally to plan the manual rotation, which would explain the training effect on mental rotation performance.

## Mental rotation tests

Many of the results reported on mental rotation tests inspired by the design of Shepard and Metzler (1971) are only analysed for the rotated but not for the mirrored stimuli. Because there is no definition of angular disparity for mirrored stimuli, a rotational strategy is not necessary to solve these trials. This is also observed in experiments such as the aforementioned study of Gardony et al. (2014). To be able to include all trials in the analysis, Jost and Jansen (2020) suggested the comparison of one stimulus figure to two base figures, which are mirrored to each other. Instead of deciding whether two figures are “same” or different,” participants have to find out whether the stimulus figure matches the left or right base figure. In their study, this task indeed produced the monotonous and approximately linear relationship between reaction time and angular disparity suggesting a rotational strategy for all stimuli. However, the slope of this relationship was steeper for answers on the left side. Moreover, Jost and Jansen also identified a steeper slope for rotations around the y-axis (picture plane) compared with rotations around the z-axis (in depth) and improvements within the test session itself, which were larger for the more difficult stimuli using larger angular disparities. The test combines aspects of traditional chronometric (Shepard & Metzler, 1971) and psychometric (Vandenberg & Kuse, 1978) tests as similar to the chronometric tests, the effect of angular disparity can be analysed on individual trials and similar to psychometric tests, always half of the answers are correct. The main advantage of this design is the increased power due to more analyzable trials and the removed need to distinguish between sensitivity and accuracy. Moreover, this design can be used for both mental and manual rotation tasks as, for example, utilised by Wohlschläger and Wohlschläger (1998), whereas manual rotation trainings using the design of Shepard and Metzler (1971) can use only congruent stimuli (Adams et al., 2014; Wiedenbauer et al., 2007; Wiedenbauer & Jansen-Osmann, 2008). By using the same design for the training, more direct comparisons between training and tests are possible. However, further research in the applicability of the design is necessary as the effects of this design have not yet been widely investigated.

## Goal of this study

Due to the lack of separation of the visual and motor component in manual rotation trainings, it is the main goal to determine which component of the manual rotation training influences mental rotation performance. Based on the mental rotation experiment of Jost and Jansen (2020) we conducted mental rotation tests and manual trainings, in which both the visual component and the congruency of the motor component were separated. The following three training conditions were investigated: the “wheel” training comprises the manual rotation of stimuli using a steering wheel (causal and congruent motor activity for visual rotation), the “button” training the manual rotation of stimuli using button presses (causal but not congruent motor activity for visual rotation), and the “visual” training the automatic visual rotation of stimuli (no causal motor activity). The following primary hypotheses are:

H1. All training conditions improve mental rotation performance.
H2. The “wheel” training shows a larger training effect than the “button” training due to the congruent motor activity and both “wheel” and “button” training show a larger training effect than the “visual” training due to the causal motor activity.

As secondary hypotheses, we investigate at first, if the effects of the test design of Jost and Jansen (2020) can be replicated and second, the relevance of gender and experience. Between participants, the effect size of gender differences varies between null or small effects (*d* = 0–0.45) in chronometric mental rotation tests and large effects (*d* > 0.7) in psychometric mental rotation tests with better performance of male participants compared with female participants (Jansen-Osmann & Heil, 2007b; Voyer & Jansen, 2016; Voyer et al., 1995). For the test of Jost and Jansen (2020) gender differences have not yet been studied. As it resembles both classical chronometric and psychometric tests, we expect at most medium effect sizes (*d* = 0.5) and we expect these to diminish in the posttest if pretest differences occur. Effects of training and previous experience are large (*d* > 0.7) and persist throughout multiple sessions (Jost & Jansen, 2020; Meneghetti et al., 2017). Thus, we expect participants with previous experience with mental rotation to perform better in both pre- and posttest.

The following secondary hypotheses are investigated:

S3. Regarding the test design: We expect an improvement of mental rotation performance over time with larger improvements for larger angular disparities. Furthermore, performance differences by the side of the correct answer and by the axis of rotation are expected (see Jost & Jansen, 2020).
S4. Regarding differences by gender and experience: Male participants will perform better than female participants. Participants with experience will perform better than participants without experience. We expect the worse performing group to improve more between pre- and posttest.

Moreover, we preplanned exploratory analyses on differences in training effects between the trained axes and the stimuli used during the training session and the pretest as well as the effects of training parameters (such as planning times and rotation speed) on posttest performance. Previous research on both mental and manual training found both improvement only on the trained axes and models, i.e., instance-based learning (Heil et al., 1998; Wiedenbauer et al., 2007) as well as transfer to unlearned models and axes, i.e., process-based learning (Adams et al., 2014; Wiedenbauer & Jansen-Osmann, 2008; Wright et al., 2008). Because the manual rotation allows further characterization by training parameters and Adams et al. (2014) found that these parameters improved differently after training, we want to explore if and how these influence mental rotation performance. This could also help to identify the aspects of the training which are most important for its effectiveness.

## Method

### Participants

While overall effects of a manual training of mental rotation are typically moderate to large compared with a control group (Adams et al., 2014: $\eta_{p}^{2}$ = .13–.18; Wiedenbauer et al., 2007: *d* = 0.66) effect sizes between different types of manual training are unknown but expected to be lower. Because repeated practice of mental rotation has shown improvements over larger training volumes (Meneghetti et al., 2017), we assumed larger effects for longer training sessions and employed a training of about twice the volume compared to Adams et al. (2014) and Wiedenbauer et al. (2007).^1^ We estimated the total required number of participants at 192 (64 per training condition) in G*power (Faul et al., 2007). This should yield appropriate power of .8 for medium effect sizes of *d* = 0.5 at the standard .05 alpha error probability for all pairwise comparisons between groups and for small effect sizes of *f* = 0.11 for the within-between interaction of groups and their improvements. This should also suffice for appropriate power regarding the within subjects effects of mental rotation for the secondary hypothesis S3. Brysbaert and Stevens (2018) suggest at least 40 participants with at least 40 trials per condition which we should exceed with our design. For the secondary hypothesis S4 regarding gender and previous experience estimated effect sizes are small to medium (*d* = 0.4) for gender and large (*d* = 0.7) for previous experience. While we did not target participants of specific gender or experience level, we expected a somewhat symmetric distribution. Assuming at least 64 participants in the smaller of the two groups in each case, G*power (Faul et al., 2007) shows sufficient power for the analysis.

Participants were recruited by advertisement in the newsletter for students (Bachelor Applied Movement Science) at University of Regensburg and received study credit for participation. They were required to physically be able to press pedals with their feet and use their hands to turn a steering wheel. Other than that, there were no exclusion/inclusion criteria.

Due to the outbreak of the COVID-19 pandemic, testing was interrupted before the targeted number of participants was reached. The global pandemic was likely to cause disruptions in education, physical, and psychological well-being as well as other effects during the large breaks in time of testing (such as an increased scientific interest in medicine or increased use of digital devices), which we expected to affect mental rotation performance and increase variance within groups but did not expect to alter the relative effectiveness of trainings. By including participants both before and after the pandemic, additional confounding variables would have to be analysed. If such a new analysis were necessary, it seems beneficial to also incorporate findings of present results to improve the experimental design and target more specific research questions. Thus, we decided to analyse and publish the results of the already tested 121 participants (“wheel” training group: *N* = 38, 12 men and 26 women, “button” training group: *N* = 42, 17 men and 25 women, and “visual” training group: *N* = 41, 11 men and 30 women). The mean age was 21.4 years (*SD* = 1.9) and did not differ significantly between groups. Data from further three participants were incompletely recorded due to electrical failures and other programmes interfering during the experiment and not analysed. The desired power for the main hypothesis is thus only achieved for effect sizes of *d* = 0.62–0.63 for pairwise comparisons and *f* = 0.14 for the within-between interaction. Nevertheless, we deemed the analysis interesting both for insight into the achieved training effects as well as modifying the experiment for future research. The use of mixed models for statistical analysis should also enhance power compared to the power analysis for *t*-tests and analyses of variances (ANOVAs). Furthermore, the additional Bayesian analysis indicates sufficient evidence for the main hypotheses.

### Material

#### Mental rotation

**Figure 1.**
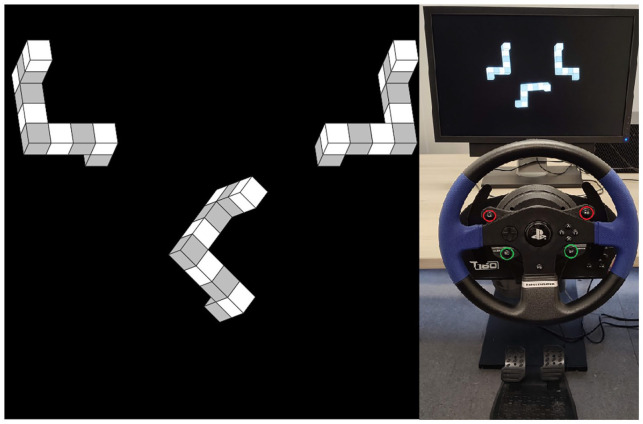
Mental rotation task and manual rotation setup. *Note.* Left: Mental rotation task. The top two figures are the base figures, which are mirrored to each other. The stimulus in the centre is a rotated version of one of the two base figures and participants are tasked to identify the congruent base figure. Right: Experimental setup including pedals and steering wheel, which participants could use to rotate the stimulus in the “wheel” and “button” condition. The buttons used are circled red (L2 and R2, upper two buttons) and green (*SE* and ST, lower two buttons).

Stimulus presentation and response handling were controlled with OpenSesame software (Mathôt et al., 2012) on a Dell OptiPlex 7050 Tower stationary desktop with a Dell P2210 screen (22,” 1600 x 1050, 60 Hz). The screen was placed approximately 40 cm from the edge of a desk with a Thrustmaster T150RS steering wheel attached centrally in front of the screen and the according two foot-pedals placed approximately 40 cm from the edge of the desk on the floor, both using the more resistant spring of the brake pedal. The pedals were placed against a metal plate to prevent movement backwards (see Figure 1). The internal forces of the steering wheel were scaled to 50% and set to return to the neutral position. This allowed turning the wheel with little force and a reliable return to the neutral position when the wheel was not held. Participants were seated in a wheeled office chair and were free to adjust their seating position.

Cube figures were used as stimuli and were generated from the stimulus library of Jost and Jansen (2020) with the parameters given in Table 1. The mental rotation task was presented according to the layout of Jost and Jansen (2020) with two base figures at the top and one stimulus figure below with a vertical shift of 150 pixels (above the centre of the screen for the base figures and below the centre for the stimulus figure) and a horizontal shift of 300 pixels (left and right of the centre of the screen for the base figures). The resulting task is shown in Figure 1. Participants’ task in this mental rotation test is to select the base figure that is congruent to the stimulus figure.

**Table 1.** Parameters for generation of stimuli.

Parameter group	Parameter	value
Colour options	Background colour	Transparent (black)
	Border colour	black
	Face colour	Grey, white
Sizing and formatting	Cube Diameter	50px
	Image size	440px*440px
	File format	png
	Centering	Optical
Model properties	Base orientations	a, b
	Models	Peters and Battista (2008), 1–16
	Base rotation angles (x, y,z)	–15°,0°,15°
	Angle difference	45°
	Rotation axes	y, z

Trials were shown until a response was given and after every trial the participants received feedback for 1000 ms (✔- right, ✘- wrong) shown at the centre of the screen at font size 40. The next trial did not start if a pedal was pressed or the wheel was turned by more than 25° during the “wheel” training condition and instead a “+” was shown at the centre of the screen until all pedals were released. The order of stimuli was block randomised at the start of every part of the experiment using 20 blocks, such that in each block, each unique combination of eligible stimulus properties occurs only once. No stimulus occurs both in the last 10 stimuli of one block and in the first 10 stimuli of the following block. Each part of the experiment was limited by time such that the maximal number of stimuli was never reached.

Participants were instructed prior to the first trial by on-screen text to press the left foot pedal with their left foot if the stimulus could be rotated into congruence with the left base figure. If the stimulus could be rotated into congruence with the right base figure, participants should press the right foot pedal with their right foot. Pedals had to be pressed a minimum of halfway down to register. Participants were asked to answer as quickly and as precisely as possible for both the pre- and the posttest but not for the training session.

In the pre- and posttest, participants were instructed to rotate the stimulus figure in their mind and select the base figure that is congruent to the stimulus figure.

In the “wheel” training condition, participants were instructed to turn the stimulus figure using the steering wheel into congruence with one base figure. The rotation of the stimulus was updated at every computational step to the position of the steering wheel rounded to the nearest 3°. Participants were instructed to release the wheel between trials to return to the neutral position.

In the “buttons” training condition, participants were instructed to turn the stimulus using the buttons on the steering wheel. They had to simultaneously press the two top buttons (L2 and R2) to turn the stimulus clockwise and the two bottom buttons (*SE* and ST) to turn the stimulus counterclockwise (Figure 1). They had to press two buttons to avoid congruence or incongruence between the pressed buttons and the base figures. The stimulus was turned 3° in every computational step as long as the buttons were pressed.

In the “visual” training condition, participants were instructed to watch the stimulus turn into congruence with one base figure. After showing the stimulus for 500 ms the stimulus turned 3° in every computational step until congruence was achieved. The direction of rotation was always the shortest path and random for starting angles of 180°. Due to a programming error, the stimulus was already turned 3° before the first showing.

As computational steps took about 60 ms on average, the rotational speed was about 50°/s in the button and visual training condition which is comparable to the mental rotational speed of Jost and Jansen (2020: 64°/s) and the manual rotation speed of Wiedenbauer et al. (2007: 43.48°/s).

In all training sessions, answers were only allowed after a discrepancy of at most 9° between the stimulus and the correct base figure was achieved at least once. Training sessions only used rotations around the y-axis (the picture plane) and no starting angles of 0°.

Reaction time, accuracy, stimulus type (model, angular disparity, rotational axis, stimulus orientation, base orientation), and time since start of each part of the experiment were recorded for all trials. During the trials the rotation of the steering wheel, the state of the relevant buttons of the steering wheel, the position of the pedals, and the shown angle of the stimulus were recorded for every computational step.

#### Demographics

A digital questionnaire was used to collect demographic information. Participants were asked about their previous experience with mental rotation (participants had to indicate if they had or had not participated in other mental rotation experiments before, yes/no), age (in years), gender (male, female, or diverse), information about their menstrual cycle, physical and musical activity, and handedness. Besides previous experience and gender, these were not part of the analyses in line with the preregistration.

### Procedure

Participants completed a pretest of 10 min followed by a training session for 30 min, a posttest of 10 min, and a digital questionnaire. Between all parts, participants had a self-paced break with instructions for the next part. Participants were informed before the start of the experiment about the length of each part and were shown again before each part. For the duration of the experiment and the questionnaire, participants were alone in the experimental room. All parts were controlled by time and not by the number of stimuli to ensure a comparable overall duration of the experiment between participants.

Participants were randomly assigned to the training conditions using block randomisation with one block generated by random sampling in *R* (R Core Team, 2018). For each of them, the base models of the stimuli were randomly selected using shuffling in OpenSesame (Mathôt et al., 2012) such that two unique models were used exclusively in each of the three parts and two unique models were used in each combination of two parts but not in the third. As a result, six different models were used in each part and 12 models were used in total for each participant. The remaining four models were not used. Models were randomly selected for every participant to avoid influences of systematic differences between models.

### Statistical analysis

The accuracy and response time of each trial were used as dependent variables and the training condition (group), the angular disparity, time (since start of each part, within each part) and block (pretest or posttest), the side of the correct answer, the rotational axis, and gender and previous experience of participants were used as independent variables. Angular disparity and time were treated as continuous variables and the categorical variables used treatment contrasts. Contrary to our preregistration the angular disparity was calculated for each rotational axis separately. This allowed us to include non-rotated stimuli, for which the rotational axis is not well defined, in the analysis of axes. Given that an improvement over time is expected and that this improvement is expected to be larger for larger degrees of rotation, the four-way interaction degree*time*group*block for each axis was analysed for the main hypotheses. Here, the effect of time represents the improvement within the pre- and posttest, whereas the effect of block describes the improvement between tests, i.e., the treatment effect. We expected an improvement by block, which exceeds the expected improvement over time for all conditions, and the interaction of block*group to explain differences in improvement between groups. For the secondary hypothesis, we analysed the interactions degree*time*side for each axis, gender*block, and experience*block.

Outliers were determined for each rotation angle by a deviance of more than three standard deviations from the mean reaction time of all stimulus pairs with the same rotation angle and were excluded from all analyses. Reaction time was additionally only analysed on correct responses. By this procedure, 1,178 of 85,354 trials (1.3%) were deemed as outliers (455 of 16,162 in the pretest, 559 of 49,119 in the training, 164 of 20,073 in the posttest). Of the remaining trials, 6,766 responses (8.0%) (2,739 in the pretest, 1,477 in the training, 2,550 in the posttest) were incorrect.

Statistical analysis was performed according to Jost and Jansen (2020) with linear mixed models using lme4 package (version 1.1-21; Bates, Mächler, et al., 2015) in *R* (version 3.5.1; R Core Team, 2018). Model parameters were estimated by maximum likelihood estimation using bobyqa algorithm wrapped by optimx package (version 2018-7.10; Nash & Varadhan, 2011) as optimizer. Model fit was calculated by using likelihood ratio tests to compare models with and without the fixed effect of interest. The resulting *p*-values were compared to a significance level of .05. Visual inspection of residual plots did not reveal deviations from homoscedasticity or normality in any model.

For the significant effects of interest, we report both the unstandardized effect sizes and confidence intervals calculated by using parametric bootstrapping with 1,000 simulations in line with recommendations of Baguley (2009) and Pek and Flora (2018). While standardised effect sizes are routinely used for power analysis and meta analyses, unfortunately there does not exist an agreed upon way to compute standardised effect sizes in linear mixed models (Feingold, 2009; Hedges, 2007; Rights & Sterba, 2019). Nevertheless, linear mixed models offer several advantages over traditional use of ANOVAs. For example, linear mixed models allow simultaneous analysis of by-participant and by-item variances and thus eliminating the need to average over participants or items, while also allowing analysis of unbalanced data and achieving higher statistical power (Barr et al., 2013; Hilbert et al., 2019).

Model building was based on the research of Barr et al. (2013), Bates, Kliegl, et al. (2015), and Matuschek et al. (2017), starting with a model with random intercepts and slopes for every appropriate fixed effect and reducing the model complexity by dropping non-significant variance components (to avoid over-parameterization at the start we included all two-way interactions for random slopes by participant and only the main effects for random slopes by the stimulus model). Non-significant fixed effects were further stepwise removed from the model, such that effects which least decreased model fit were removed first and a model containing only significant fixed effects remained. Non-significant effects were then tested for an improvement of model fit by inclusion in the resulting model, while significant effects were tested for worsening of model fit by exclusion of the effect. The resulting models for each parameter are described in the results section. The analysis of numerical main effects contained in significant interactions was performed according to Levy (2014). Degree was centred such that main effects show the average improvement over all angles. Time was normalised such that time 0 was set to the end of the pretest and the start of the posttest. As a result, the effect of block represents the difference between the estimated end of the pretest compared to the beginning of the posttest. While the comparison between the groups was the main goal of the study, this inclusion of time in the analysis allows for better control of practice effects within the tests. Thus, the treatment effect of interest are the block*group interactions.

Due to the non-significance of many results we have retrospectively calculated Bayes factors to distinguish evidence in favour of no effects (Dienes, 2014; Wagenmakers, 2007). Due to the retrospective nature of the analysis we opted to calculate Bayes factors objectively based on the approximation of Wagenmakers (2007) and compared it to the decision boundary factor 3 or 13 . We do note a monotonous relationship between the Bayes factors and *p*-values and thus the necessity to consider the Bayes factors also for significant results, which we elaborate on in the supplementary material.

For both frequentist and Bayesian analyses there is ongoing discussion about the optimal procedure and we release all data and code in accordance with the suggestion of Matuschek et al. (2017).

## Results

### Descriptive statistics

**Figure 2.**
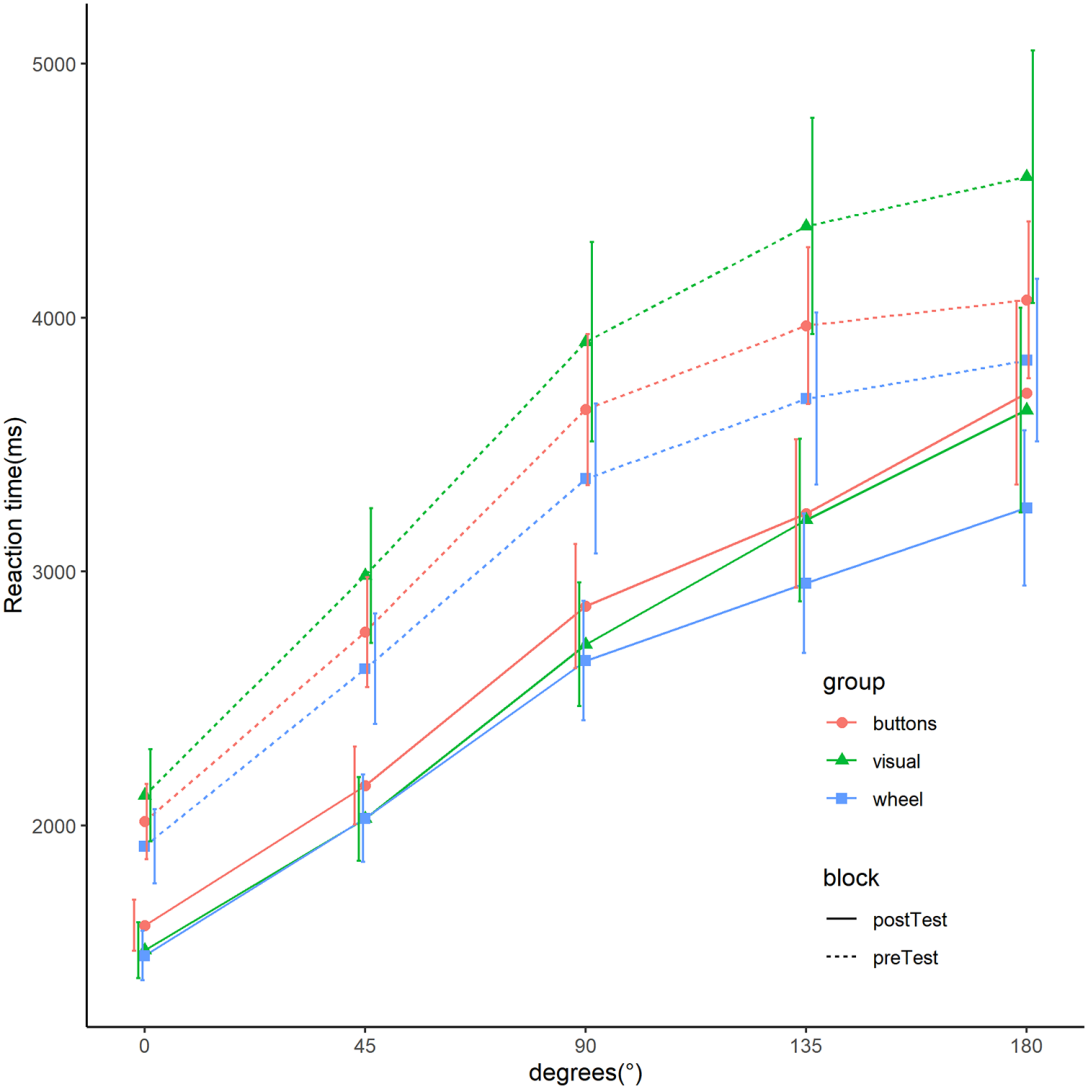
Mean reaction time of mental rotation trials as a function of angular disparity for the three groups and two tests. *Note.* Mean reaction time is calculated for all correctly answered trials of every participant and then averaged over all participants. Error bars show 95%CIs computed by ggplot2 (Wickham, 2016).

**Figure 3.**
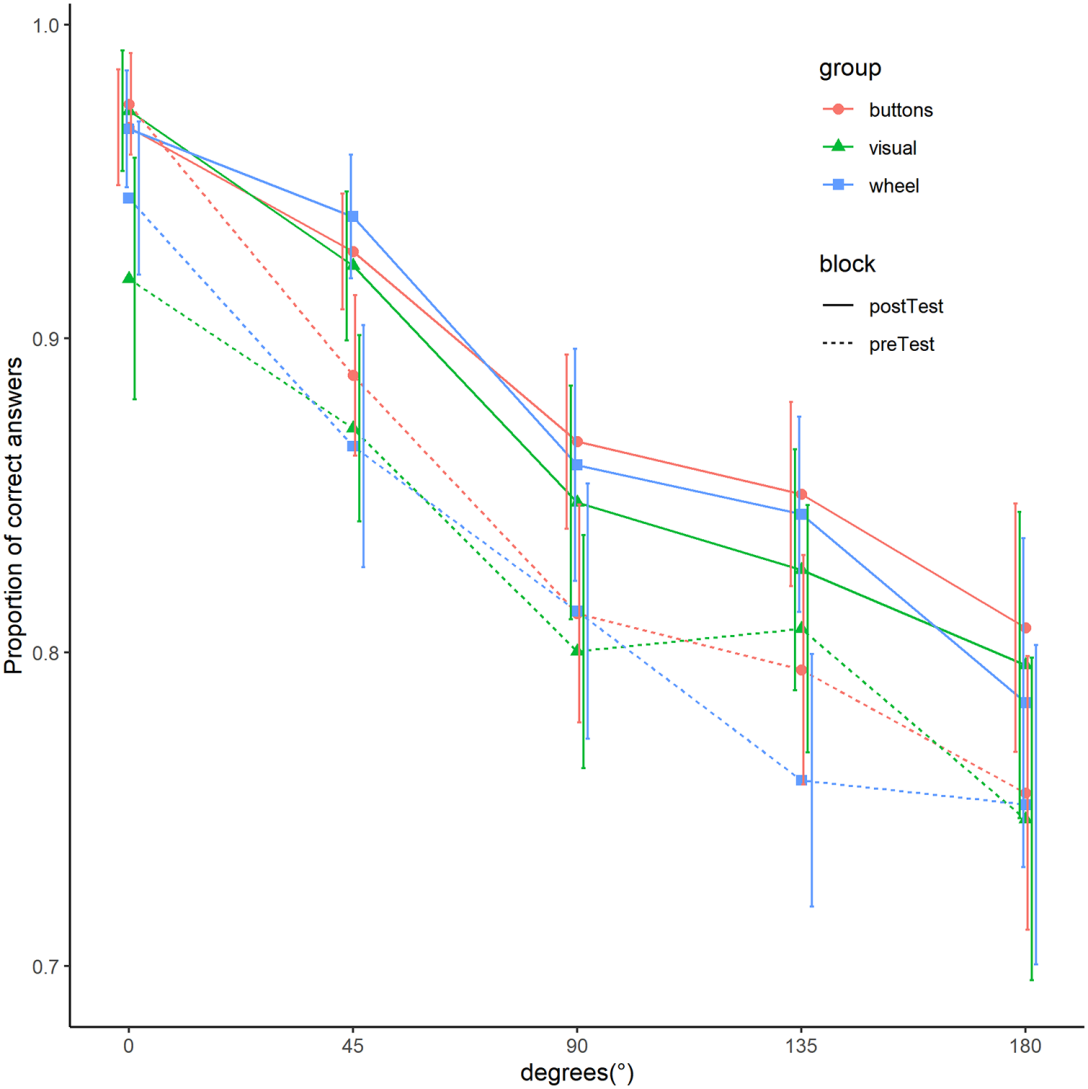
Mean accuracy of mental rotation trials as a function of angular disparity for the three groups and two tests. *Note.* Mean accuracy is calculated for every participant and then averaged over all participants. Error bars show 95%CIs computed by ggplot2 (Wickham, 2016).

Summarised performance data are shown for reaction time (Figure 2) and accuracy (Figure 3). Due to the time-controlled nature of the experiment, participants finished different number of mental rotation trials. To account for this, mean data are first calculated for every participant and then averaged over participants. Further summaries of behavioural data and summarised demographic data can be found at https://github.com/LeonardoJost/MMR.

### Comparison of pre- and posttest

#### Reaction time

For the analysis of reaction time, model building resulted in a model with random intercepts and random slopes for degree, time (since start of each part), and block by participant and random intercepts by model. Significant effects were found for degree(y-axis)*time*block*group, degree(z-axis)*block, degree*side, degree*time, gender*block, and experience*block.

Overall and in line with hypothesis H1, there was an improvement for all stimulus properties (degree and axis), which only differed in magnitude. This was supported by both frequentist and Bayesian analysis. The significant four-way interaction was inconclusive regarding the Bayes factors suggesting possible overall differences between groups in learning over time of rotations around the y-axis within the pre- and posttest. However, no partial interactions containing the block*group interaction proved significant contrary to hypothesis H2. The Bayes factors indicated strong evidence for no effects. This suggests a comparable overall treatment effect (the block*group interaction), comparable overall improvements of rotations around the y-axis (the deg[y]*block*group interaction), and comparable changes of learning within tests (the time*block*group interaction) between groups.

In line with our secondary hypothesis S3 improvements over time were larger for larger angles and answers on the right side showed a lower slope by degree compared with answers on the left side but no main effect between the sides proved significant. The Bayes factors were in support of the significant results and suggested null effects for the non-significant results. The frequentist and Bayesian analysis disagreed on the difference in improvements over time between axes, suggesting the necessity for further research. Contrary to the secondary hypothesis S4, neither main effects of gender nor experience were significant but men improved more than women and participants without experience improved more than participants with previous experience. The Bayesian analysis supported the larger improvement for men and suggested no overall gender differences but required more evidence for both effects of experience.

Regarding the exploratory comparison of training effects between axes, rotations around the z-axis showed a steeper slope by degree at the start of the posttest compared to the end of the pretest, whereas there was no significant difference for the y-axis. This was partially supported by the Bayes factors, requiring more evidence for the z-axis. These results partially suggest a larger training effect for rotations around the y-axis but due to the overall improvement between blocks also a training effect for the z-axis.

For the interactions by block, we conducted separate analyses for the pre- and posttest. Participants with experience were significantly faster in the pretest but not in the posttest. Despite the difference in training effect, gender differences were not significant, in neither pre- nor posttest. The decomposition of the four-way interaction revealed significant axis differences in the pretest and between-group differences regarding the axes and improvements over time in the posttest. Bayes factors were inconclusive regarding the effect of experience and differences in improvement over time in the posttest. For the between-group differences regarding the axes, the Bayesian analysis contradicted the frequentist analysis suggesting evidence of no differences. (see Table 2).

**Table 2.** Statistical analysis of reaction time.

Variable	Estimate	*SE*	Test statistic	*p*	95% CI	BF
Intercept	2796.21	118.16			2562.43, 3007.02	
Deg(y)*time*block*group			χ²(2) = 8.17	.017		2.04
Deg(y)*block*group			χ²(2) = 0.41	.816		98.73
Time*block*group			χ²(2) = 2.04	.361		43.64
Block*group			χ²(2) = 2.05	.359		43.40
Block(pre-post)	242.91	52.29	χ²(1) = 13.44	< .001	141.46, 351.74	0.01
Deg*time*side			χ²(1) = 3.71	.054		1.72
Deg(y-z)*time			χ²(1) = 3.90	.048		1.56
Deg(z)*time	–364.65	149.92	χ²(1) = 5.91	.015	–649.91, –63.27	0.57
Deg*side(right-left)	–107.42	26.00	χ²(1) = 17.06	< .001	–157.13, –55.48	< .01
Side (right-left)	10.03	13.41	χ²(1) = 0.56	.455	–16.66, 36.23	8.32
Block (pre-post)*deg(y)			χ²(1) = 0.31	.578		9.42
Block (pre-post)*deg(z)	–133.02	56.60	χ²(1) = 5.52	.019	–243.76, –14.31	0.70
Block (pre-post) *gender(female-male)	–246.34	76.52	χ²(1) = 9.70	.002	–403.35, –103.40	0.09
Block (pre-post) *Experience (no-yes)	338.13	132.44	χ²(1) = 6.14	.014	67.27, 609.69	0.51
Gender (female-male)	28.17	75.81	χ²(1) = 0.02	.875	–107.39, 184.71	10.87
Experience (no-yes)	210.18	131.65	χ²(1) = 3.71	.054	–57.25, 467.33	1.72
**Pretest**						
Gender (female-male)	–153.83	111.62	χ²(1) = 1.81	.178		4.44
Experience (no-yes)	643.10	199.01	χ²(1) = 9.58	.002	216.62, 1002.14	0.09
Deg (y-z)	61.88	20.99	χ²(1) = 8.68	.003	21.42, 102.42	0.14
**Posttest**						
Gender (female-male)	–35.01	70.17	χ²(1) = 0.25	.620		9.71
Experience (no-yes)	204.56	120.93	χ²(1) = 2.77	.096		2.75
Time*group (buttons)	–463.20	276.12	χ²(2) = 8.79	.012	–1004.78, 67.88	1.49
Time*group (visual-buttons)	–1176.73	390.80			–1921.63, –425.25	
Time*group (wheel-buttons)	–647.79	397.14			–1394.02, 138.12	
Deg(y-z)*group (buttons)	–216.04	26.03	χ²(2) = 6.50	.039	–268.58, –161.78	4.69
Deg(y-z)*group (visual-buttons)	77.82	36.93			6.09, 149.31	
Deg(y-z)*group (wheel-buttons)	–8.01	37.00			–81.57, 65.68	
**Exploratory**						
*N*(pretest)*group			χ²(2) = 4.27	.118		14.33
*N*(pretest)	–8.09	0.76	χ²(1) = 51.79	< .001	–9.51,–6.52	< .01
*N*(training)*group(buttons)	–2.57	1.10	χ²(2) = 7.85	.020	–4.65, –0.39	2.39
*N*(training)*group(visual-buttons)	–2.96	1.54			–6.19,–0.08	
*N*(training)*group(wheel-buttons)	0.60	1.19			–1.69, –2.71	
*N*(training)			χ²(1) = 23.57	< .001		< .01
Proportion(short direction)*group(buttons)	410.19	392.36	χ²(2) = 5.00	.025	–346.79, 1200.04	9.93
Proportion(short direction)*group(wheel-buttons)	–1650.71	726.36			–3010.83, –188.41	
Trained models(new-old)	–59.46	17.39	χ²(1) = 11.69	< .001	–91.02, –24.83	0.03
Trained models(pretest-training)			χ²(1) = 0.00	.986		11.00
Trained models*group			χ²(2) = 3.31	.191		23.12

Note. The values for degree and time (since start of each part) represent estimated changes corresponding to changes of 100° and 30 minutes of testing time. BF stands for the approximation of the Bayes factor by Wagenmakers (2007) in favour of the null hypothesis. *SE*: standard error; CI: confidence interval.

#### Accuracy

Accuracy was analysed by a general linear-mixed model, which used a binomial distribution. Model building resulted in a model with random intercepts and random slopes for degree, time (since start of each part), and degree*time by participant and random intercepts and random slopes for degree and time by model. Significant effects were found for time*block, degree (y-axis)*time*group, and degree (z-axis)*block.

As in the analysis of reaction time, this suggests differences between groups in learning within the tests but no differences in the treatment effect between tests. Overall, there was an improvement from pre- to posttest but again, the rotation around the z-axis showed a steeper slope by degree. Improvements over time were only significant in the pretest and did not differ significantly by degree. No effects of gender or experience were significant. The Bayes factors were in support of the significant results and suggested null effects for the non-significant results except for the inconclusive interaction of gender and block (see Table 3).

**Table 3.** Statistical analysis of accuracy.

Variable	Estimate	*SE*	Test statistic	*p*	95% CI	BF
Intercept	2.32	0.14			2.04, 2.61	
Block*group			χ²(2) = 5.36	.069		8.30
Deg*time*side			χ²(2) = 1.81	.404		48.95
Deg*time			χ²(1) = 0.21	.644		9.90
Deg*side			χ²(1) = 1.72	.190		4.65
Time*block(pre-post)	1.76	0.33	χ²(1) = 27.85	<.001	1.15, 2.39	< .01
Block(pre-post)*deg(z)	0.36	0.07	χ²(1) = 26.84	<.001	0.23, 0.49	< .01
Block(pre-post)	–0.25	0.06	χ²(1) = 15.37	<.001	–0.38, –0.13	< .01
Time*deg(y)*group			χ²(2) = 12.42	.002		0.24
Gender*Block			χ²(1) = 4.03	.133		1.47
Gender(female-male)	0.09	0.13	χ²(1) = 0.47	.493	–0.17, 0.34	8.70
Experience*Block			χ²(1) = 0.55	.761		8.36
Experience(no-yes)	0.01	0.22	χ²(1) = 0.00	.962		10.99

*Note.* The values for degree and time (since start of each part) represent estimated changes corresponding to changes of 100° and 30 min of testing time. BF stands for the approximation of the Bayes factor by Wagenmakers (2007) in favour of the null hypothesis. *SE*: standard error; CI: confidence interval.

Regarding the hypotheses, the results for accuracy were either in the same direction as the results for reaction time or supported null effects. This suggests that the changes in reaction time are not due to reaction time—accuracy tradeoffs.

### Exploratory analysis

We performed explorative analyses on the influence of training performance on posttest performance. To begin, we defined and computed metrics to characterise the training performance for which we provide descriptive statistics overall and for the changes during the training session. Subsequently, we have analysed the relationship between these metrics and posttest performance to explore possible connections.

#### Description of the training session

**Figure 4.**
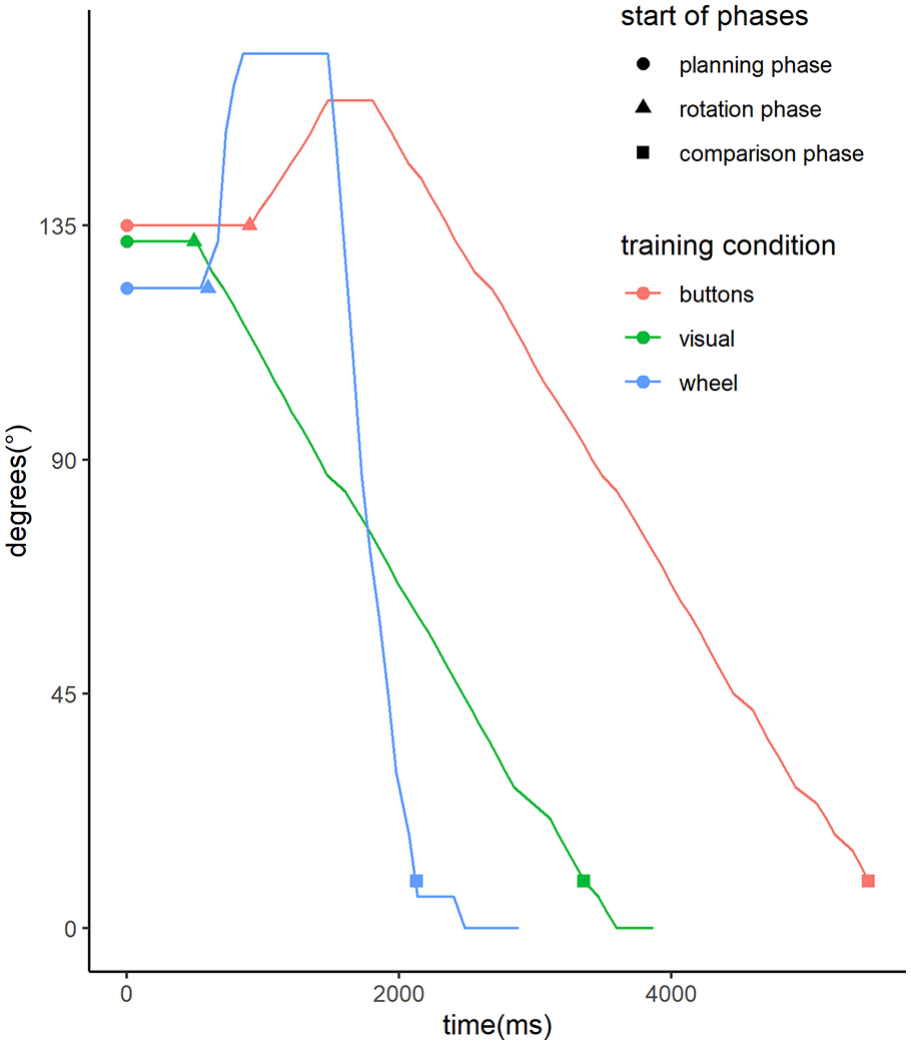
Exemplary movement of an object during training trials. *Note.* The graph depicts one example of a training trial for each condition for a starting angle of 135°. The calculated starts of each phase are marked for each trial. The planning phase starts with the trial onset. The rotation phase starts just before the first angular displacement. The comparison phase starts once a deviation of at most 9° from the target is reached for the first time. The trial ends once an answer is recorded. There are some small fluctuations in the calculated times and the slopes in the “visual” and “buttons” training due to fluctuations in the display frame rate. Because the wheel was not always perfectly aligned and the visual rotation started already turned by 3°, there are small differences in the starting angle.

**Figure 5.**
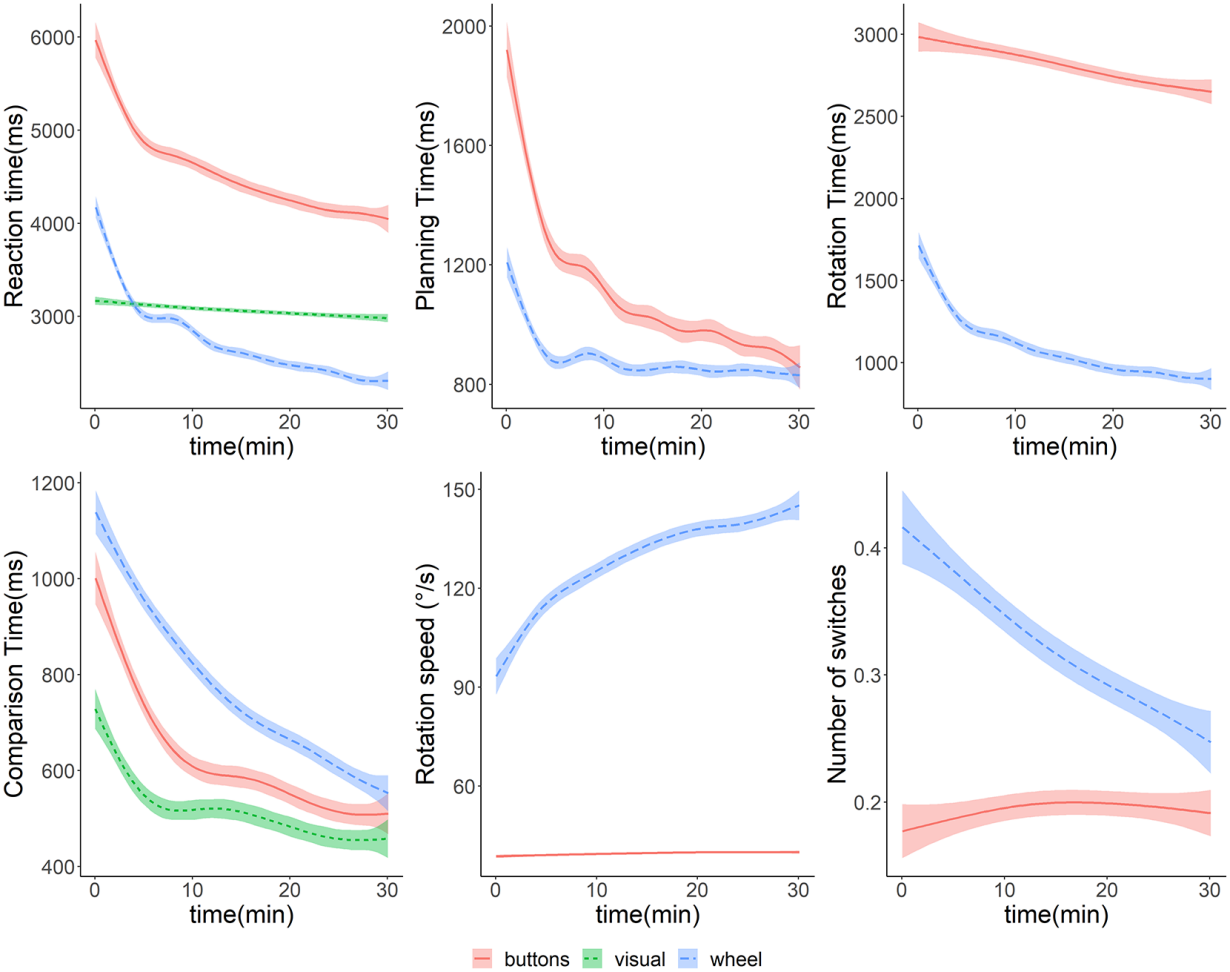
Parameters of the training session separated by groups and their changes over time. Note. From left to right and top to bottom: (1) reaction time, (2) planning time, (3) rotation time, (4) comparison time, (5) rotation speed, and (6) number of switches of rotation direction. Smoothed conditional means over time are generated using a generalised additive model in ggplot2 (Wickham, 2016).

First, the average accuracy for the training trials was above 95% for all three groups and all starting angles, which is comparable to the accuracy of non-rotated trials in the mental rotation test. Next, we have looked at the overall reaction time and other parameters of the training session. Similarly to Adams et al. (2014), we have divided each trial into three phases: A planning phase, a rotation phase, and a comparison phase (see Figure 4 for examples). The comparison phase differs from the fine tuning phase of Adams et al., as they required participants to match figures as closely as possible whereas our participants were asked to select one of two comparison figures. The planning phase describes the time from the start of the trial to the first angular deviation from the starting position. To account for random fluctuations possibly from returning the wheel to neutral position after the previous trial, the most common angle of the first five measurements (about 240 ms) was used and deviations were measured afterwards. The following rotation phase ends when the rotated figure is closer than 10° to the congruent base figure as answers by participants were allowed after this time. The comparison time is the further time until an answer was given. The rotation phase is further described by three parameters: the average rotation speed, the number of switches of the direction of rotation, and whether the overall rotation was performed in the shorter direction (for starting angles differing from 180°). For all parameters, we have descriptively looked at group differences over the course of the training session (Figure 5). There are differences between groups regarding the phases, both in average values as well as in changes over the course of the session. Notably, overall reaction time and most phases (except for the rotation phase of the “buttons” training and the comparison phase of the “wheel” training) show a steeper decline in the first 5 min compared with the following time in all groups. Regarding rotations in the short direction, average values were 85% for the “buttons” group and 89% for the “wheel” group. This suggests that the planning and rotation phases were used to determine the shortest path of rotation and mental comparison processes are performed before the comparison phase.

#### Analysis of training effects

To analyse the training effects of the training parameters, we compared all training parameters averaged by participant and their interaction with group on posttest performance of reaction time. As the training parameters are linked to average performance, we further compared the significant training parameters with the overall number of trials in both the pretest and the training session. Both the number of pretest trials and the number of training trials as well as the interaction of the number of training trials with group were significant. With increasing number of pretest trials and increasing number of training trials the reaction time in the posttest decreased. For the number of training trials, this effect was larger in the visual training group compared with the other groups. Regarding the training parameters, the proportion of rotations in the short direction, and the interaction of the proportion and group showed significant effects on posttest performance. The proportion of rotations in the short direction showed a positive relationship with reaction time in the buttons training group and a negative relationship in the wheel training group. The Bayes factors were in support of the effects regarding the number of pretest trials but inconclusive regarding the number of training trials and contradicting regarding the proportion of rotations in the short direction.

In a second analysis, we compared the posttest performance of models used in the pretest and in the training session with models only used in the posttest. There was no significant difference between models used in the pretest and in those used in the training session but both showed significantly faster reaction times than the new models in the posttest. As the differences between models were smaller than differences between blocks, this suggests a transfer of training effect to the new models. Differences between groups were not significant. The Bayes factors were in support of the significant results and suggested null effects for the non-significant results (See Table 2).

## Discussion

In this study, we provide insight into the training effects of manual rotation movements on mental rotation tasks. In line with previous research on manual training of mental rotation (Adams et al., 2014; Wiedenbauer et al., 2007; Wiedenbauer & Jansen-Osmann, 2008) our results show an improvement in mental rotation performance from manual training, but these improvements do not differ between a rotational and a non-rotational movement. Moreover, by isolating the component of concurrent visual rotation of stimuli our experiment provides evidence that it is not the motor activity but the concurrent visual rotation that leads to improvements in mental rotation tasks. As repeated mental rotation tasks, where a visual rotation of stimuli in the mind is assumed, show comparable improvements to manual rotation tasks (Adams et al., 2014), this implies that the visual rotation whether internal (imagined) or external (physical or visualised) is the main reason for improvements in mental rotation tasks. The importance of the sensory output is in line with the study of Janczyk et al. (2012). Despite the presentation of visual rotations, the mental representation of this process is not necessarily visual similar to the identified nonvisual representation of mental rotation (Ilan & Miller, 1994; Jansen-Osmann & Heil, 2007a; Liesefeld & Zimmer, 2013).

### Training effects in mental rotation

While overall differences between training groups were not significant, a more detailed analysis revealed possible differences in the improvements within the posttest of the slope of reaction time by angle. These might be caused by different parts of the mental rotation process being trained by the training conditions and the repeated mental rotation tasks in the posttest.

Improvements in manual rotation tasks on the other hand might also be driven by familiarisation with the motor behaviour, which is not trained by the mental rotation tasks. This can be seen by the steep decline in reaction time in the first 5 min of the training session in both manual training groups despite the preceding mental rotation tasks. Improvements afterwards are more in line with improvements found by repeated mental rotation tasks (Jost & Jansen, 2020). This supports the results of Adams et al. (2014) that manual rotation performance is improved more by practicing manual rotation tasks than by practicing mental rotation tasks and might also pose a solution for the conflict between their results and the common process hypothesis of Wohlschläger and Wohlschläger (1998).

Regarding the transfer of training effects on new figures, our results show better performance on previously trained figures and a comparable transfer in all groups. This result could be interpreted in support of instance-based learning but the differences between new and old models were much smaller than differences between blocks suggesting also process-based learning. The non-significant point estimates of the group differences might indicate a better transfer to new figures with increased motor activity in support of the findings of Adams et al. (2014) but this was not supported by Bayes factors, which showed strong evidence for no effects. Usage of larger differences between stimuli to facilitate these effects might be necessary to generate different transfer effects between objects due to motor activity. Regarding the transfer to untrained rotation axes, our results also show significant improvements for the untrained rotation in depth around the z-axis in all groups. In contrast to the trained axis, this improvement is characterised by a larger improvement on smaller angles. This suggests the transfer of only non-rotational parts of the mental rotation process to the untrained axis.

### Performance during training and influence on posttest performance

Our exploratory analysis of the performance in the training sessions shows that most performance parameters improve during the training and the decreasing reaction times during the training cannot be explained by a single parameter. The performance in the posttest, however, was only significantly influenced by the number of trials, both in the training session and in the pretest. The time spent on trials in the visual training group is mostly programmatically controlled and participants could only influence this time by their response time after stimuli were rotated into congruence. Thus, one could expect that the number of training trials would not be correlated with mental rotation performance in the visual training group or be less correlated than in the other groups. This was not supported in the results, indicating that the time spent on comparing and selecting identical figures is a significant part of mental rotation performance. By another account, the reaction time of the visual training trials might also be influenced by participants rotating stimuli faster mentally than the visual presentation and comparing the stimuli before congruence is achieved. Similarly to the large proportion of rotation in the shorter direction in the manual training groups, this could indicate that mental rotation and comparison processes are performed throughout the training tasks. As Adams et al. (2014) found similar training effects for mental and manual rotation interventions, the facilitation of mental processes by the training could be the most relevant for improvements.

As the reaction time is mostly independent of performance in the visual training, this type of training could be expected to be more suited to slow performers whereas the congruent manual training might be more suited to fast performers, but this hypothesis was also not supported by the results. Pretest performance was a significant predictor of posttest performance, but the Bayes factors indicated no differences between groups.

### Implications for mental rotation training

The results suggest the effectivity of a purely visual training to enhance mental rotation performance. This type of training is easy to implement and can easily be adapted for large groups or online training without the need for special equipment or motoric requirements. Such a training can be employed to boost mental rotation ability and spatial ability in general, if such a transfer were found.

Regarding the choice of how to conduct training sessions, the non-congruent manual rotation and the visual rotation would allow further parameterization. If the largest similarity with the congruent manual rotation is desirable, the choice of rotation speed and the starting time for the visual rotation group in our experiment are too low. Furthermore, we observed differences between the congruent and non-congruent manual rotation groups regarding the starting time and the number of switches in rotation direction, which might be caused by the accessibility of the buttons and the simplicity of switching directions. As the improvements were comparable between groups despite these differences, further research is necessary to understand the relationship between the parameterization of the training sessions and their training effects. This also offers the possibility to further optimise and individualise the training.

Compared with repeated mental rotation training, an advantage could be the high accuracy even for complex stimuli. As children have been shown to profit from mental rotation training starting from a young age (Fernández-Méndez et al., 2018) but suffer from the complexity of stimuli (Hoyek et al., 2012), a visual training could help accustom them to more complex tests. However, as Adams et al. (2014) found a similar effect of mental and manual training, the comparison of visual training and repeated mental rotation tasks should be investigated further.

### Evaluation of the mental rotation test

For the analysis of the mental rotation design proposed by Jost and Jansen (2020), our results confirm their proposed similarity to the original chronometric design of Shepard and Metzler (1971) but also the small left-right differences found in the original study. Reaction time increases and accuracy decreases with degree for both sides and axes and improvements are larger for larger degrees. In support of the need for further research, we also confirmed systematic differences between axes even in the pretest and small differences in the slope between answers on the left and right side. While we did find a significantly larger training effect for men, the non-significance of gender effects in both pre- and posttest is in line with small or non-existent gender differences in chronometric mental rotation tasks (Jansen-Osmann & Heil, 2007b). This was also supported by the Bayes factors suggesting no effects. As expected, the broad measure of previous participation in mental rotation experiments was an indicator of improved performance. The non-significance in the posttest can be explained by the unbalanced distribution of experience compared with our hypothesis. In line with this, the Bayes factors required more evidence for the training effects and posttest performance differences regarding experience. For both gender and experience, the worse performing group in the pretest improved more and reduced the difference to non-significance.

## Limitations

The study is limited by the fact, that there was a different number of trials in the three training groups, though overall time spent was controlled. While it is not clear if time or number of trials is more influential for training effects, this could limit the comparability between groups, which handled different number of trials, and to other studies, which use a fixed number of trials. Due the limited total time and breaks between trials, participants who solved more trials actually spent less time with the stimuli themselves. If the time spent on tasks were the main driver of training effects, our training could have benefitted slower participants more.

Another possible limitation is the fact that the visual training condition is passive, whereas the other two are active. This seems necessary for the separation of the manual and visual components but could interact with training effects.

Furthermore, we could not test 192 participants due to the pandemic interruption, as calculated from the A-priori G*Power analysis. At least for the main hypotheses this should not be a concern as indicated by the Bayesian analysis. The possible training effects with regard to previous experience should be treated with caution due to the skewed distribution of participants.

## Conclusion and outlook

This study clearly provides evidence that the visual rotation whether internal or external is the most important component for improvements in mental rotation tasks. To isolate the visual component, further investigation of the opposite direction is also necessary. The first step towards this could be the removal of visual rotation in manual rotation tasks, which should result in no or minor improvement of mental rotation performance. While our results support previous findings of manual training of mental rotation (Adams et al., 2014; Wiedenbauer et al., 2007), the design used here is more comparable to the mental rotation task as it employs a congruency judgement similar to the task used by Wohlschläger and Wohlschläger (1998).

One next step could be to analyse the different phases in the manual rotation trials more deeply to infer where differences in the mental rotation process occur. This means in the perceptual stages (perceptual processing, identification and discrimination of stimuli, identification of orientation), stages of the rotation process itself (mental rotation, judgement of parity), and the decision processing stages (response selection, execution) (Heil & Rolke, 2002). Furthermore, investigating the cognitive mechanism implicated in mental rotation task, as for example working memory could help to analyse the processes in visual and motor rotation in more depth. Hyun and Luck (2007) demonstrated that object working memory system but not the spatial working memory system provides the buffer for the storage of the objects in mental rotation tasks. Here, the influence of the visual components of the stored objects could be investigated in more detail. Another interesting point for future research might be the investigation of differences in learning over time and ceiling effects caused by different combinations of manual, mental, and visual rotation tasks and how training interventions affect the use of different rotation strategies. For this, one could ask participants for the use of their strategy between the pre- and post-test as well as the training sessions or employ other measures of strategies.

Moreover, the study has practical implications for the enhancement or the prevention of a decline in spatial abilities associated with old age (Jansen & Heil, 2009; Meneghetti et al., 2018): A visual training of mental rotation performance might be just as effective as a mental or motor training but the transfer to older adults and other spatial abilities has to be investigated further. Similarly, such a training could be employed for immobile persons such as children with spina bifida who already suffer from reduced mental rotation performance and where a manual training has proven to be effective (Wiedenbauer & Jansen-Osmann, 2007).

To conclude, this study provides evidence regarding the importance of the visual component in mental rotation tasks and also the similarity between mental and manual rotation. However, the “common process” is far from being understood from an experimental point of view.

## Supplemental Material

sj-docx-1-qjp-10.1177_17470218211039494 – Supplemental material for Manual training of mental rotation performance: Visual representation of rotating figures is the main driver for improvements Click here for additional data file. Supplemental material, sj-docx-1-qjp-10.1177_17470218211039494 for Manual training of mental rotation performance: Visual representation of rotating figures is the main driver for improvements by Leonardo Jost and Petra Jansen in Quarterly Journal of Experimental Psychology

## Notes

1. We estimated about 360 trials per participant in the 30-min training session based on an average reaction time of 4 seconds, and 1 second break between stimuli compared with 176 and 192 training stimuli.
