# Supplementary material for: Manual training of mental rotation performance: Visual representation of rotating figures is the main driver for improvements
Source: Q J Exp Psychol (Hove). 2021 Aug 13;75(4):695–711. doi: 10.1177/17470218211039494 (PMC8915228; doi:10.1177/17470218211039494)
Supplement: sj-docx-1-qjp-10.1177_17470218211039494 – Supplemental material for Manual training of mental rotation performance: Visual representation of rotating figures is the main driver for improvements [file sj-docx-1-qjp-10.1177_17470218211039494.docx]

Supplementary material for:

**Manual Training of Mental Rotation Performance: Visual Representation of Rotating Figures is the Main Driver for Improvements**

Leonardo Jost and Petra Jansen

**Comparing BIC-based Bayes factors to a model generation using likelihood ratio tests**

For the random effects structure of the linear mixed model we have mainly followed the approach of Bates et al. (2015), who also showed that Bayesian modeling supports the same random effects structure. For the fixed effects, we have reduced the model using likelihood ratio tests (LRT) to eliminate non-significant effects at the significance level $\alpha=.05$. Assuming that it is sensible to use the same models for the computation of Bayes factors we get the following relationship between the p-values and Bayes factors:

For the comparison of two models $m_{1}$ and $m_{0}$, where $m_{0}$ is nested within $m_{1}$ ($m_{0}$ typically represents the null hypothesis and $m_{1}$ a model containing $m_{0}$ and additionally a fixed effect of interest), a likelihood ratio test is based on

$$\chi^{2}\left( m_{1},m_{0} \right)=-2\left( \log L_{0}-\log L_{1} \right),$$

where $L_{i}$ is the maximum likelihood for model $m_{i}$ (and $\log L_{i}$ is the logarithmic likelihood of the model). Wagenmakers (2007) derives an approximated Bayes factor (in favour of model $m_{0}$) for the comparison of these two models as

$$\mathrm{BF}_{01}\approx\exp\left( \frac{\Delta\text{BIC}_{\text{10}}}{2} \right),$$

Where BIC is the Bayesian information criterion and

$$\Delta\text{BIC}_{\text{10}}= \text{BIC(}m_{1})-\text{BIC}(m_{0})$$

As

$$\text{BIC}\left( m_{1} \right)=-2log L_{1}+\log\left( n \right)*df(m_{1})$$

$\mathrm{BF}_{01}$ can be computed by

$$2log \left( \mathrm{BF}_{01} \right)=\Delta\text{BIC}_{\text{10}}$$

$$= \text{BIC(}m_{1})-\text{BIC}\left( m_{0} \right)$$

$$=-2log L_{1}+\log\left( n \right)*df\left( m_{1} \right)-\left( -2\log L_{0}+\log\left( n \right)*df\left( m_{0} \right) \right)$$

$$=-\chi^{2}\left( m_{1},m_{0} \right)+\log\left( n \right)(df\left( m_{1} \right)-df\left( m_{0} \right))$$

Or

$$\mathrm{BF}_{01}=\exp({(-\chi}^{2}\left( m_{1},m_{0} \right)+\log\left( n \right)\left( \Delta df\left( m_{1},m_{0} \right) \right))/2)$$

Note that $\Delta df\left( m_{1},m_{0} \right)=df\left( m_{1} \right)-df\left( m_{0} \right)$, which is the number of degrees of freedom introduced by the effect in question.

As $n$ is fixed within an experiment, there is a monotonous relationship between $\mathrm{BF}_{01}$ and χ². As χ² is again monotonously related to p by the χ²-distribution, there is a monotonous relationship between $\mathrm{BF}_{01}$ and p^[[1]](#footnote-1)^. For the cutoffs of 3 (favouring the null hypothesis) or $\frac{1}{3}$ (favouring the alternative hypothesis) for the Bayes factor we can thus calculate for a given $\Delta df\left( m_{1},m_{0} \right)$ for which values of χ² it is achieved. In our case $n=121$ (“In such a hierarchical or multilevel design, it is not quite clear what n should be. In this case, the standard choice is to take n to be the number of subjects.” Wagenmakers, 2007, p.798) and $\log\left( 121 \right)\approx4.796$. Thus for effects adding one degree of freedom (i.e. all dichotomous and numerical independent variables) we get that

$$\mathrm{BF}_{01}<\frac{1}{3}\leftrightarrow\chi^{2}\left( m_{1},m_{0} \right)>6.993\leftrightarrow p<.008$$

$$\mathrm{BF}_{01}>3\leftrightarrow\chi^{2}\left( m_{1},m_{0} \right)<2.599\leftrightarrow p>.107$$

For effects with two degrees of freedom (in our case only the independent variable group and all interactions containing it) we get

$$\mathrm{BF}_{01}<\frac{1}{3}\leftrightarrow\chi^{2}\left( m_{1},m_{0} \right)>11.789\leftrightarrow p<.003$$

$$\mathrm{BF}_{01}>3\leftrightarrow\chi^{2}\left( m_{1},m_{0} \right)<7.395\leftrightarrow p>.025$$

As a result, the Bayes factor strongly supports the null hypothesis in almost all cases where p>.05 and the null hypothesis is not rejected under the frequentist approach. In the other direction, the Bayes factor deems some significant results as inconclusive and for two degrees of freedom even favours the null hypothesis. Indeed, this is somewhat in line with Wagenmakers (2007) who in figure 6 shows the possibility of a significant result under frequentist statistics and the Bayes factor favouring the null (see also the Jeffreys-Lindley paradox; Lindley, 1957).

Reviewing the model generation using the LRT where we eliminated non-significant effects, we can now compare it to a model generation using the Bayes factor with a threshold of 1 (i.e. always sticking with the more probable hypothesis). A unique threshold is needed, as effects can only be excluded or included and there can be no region where more data is needed (as for $\frac{1}{3}<\mathrm{BF}_{01}<3$). Using the approximation of Wagenmakers (2007) this is equivalent to choosing the lowest BIC as $\exp\left( 0 \right)=1$. That is, if for any two models $\mathrm{BF}_{01}<1$ then $\Delta\text{BIC}_{10}<0$ and if $\mathrm{BF}_{01}>1$ then $\Delta\text{BIC}_{10}>0$. Choosing the model with lowest BIC is thus equivalent to removing all effects for which $\mathrm{BF}_{01}>1.$ Thus, the model selections by LRT or BIC (or AIC) are equivalent up to a different p-value cutoff. While most are arbitrary, the choice of BF=1 does distinguish between the most likely effects. For LRT, α can be chosen arbitrarily. The AIC is equivalent to an LRT-based model selection using α≈0.157 (Matuschek et al., 2017). For the BIC the equivalent α depends on n. For a more thorough comparison cf. also Lewis et al. (2011).

Going back to the relationship between BF and p, we can get the following situation:

Assume an experiment and effects of one degree of freedom (i.e. either dichotomous or numerical). Assume an arbitrary significance level α (typically .05) and an arbitrary Bayes factor cutoff $\mathrm{BF}_{0}$ in favour of the null hypothesis (typically 3). Due to the relationship between p and BF we can calculate an $n_{0}$, such that for all effects with $p>\propto$ we get $BF>\mathrm{BF}_{0}$ and for all effects with $p<\propto$ we get $BF<\mathrm{BF}_{0}$. Moreover, for all $n>n_{0}$ we get for all effects with $p>\propto$ that $BF>\mathrm{BF}_{0}$, while the other direction is maintained for all $n<n_{0}$.

For $\propto=.05$ and $\mathrm{BF}_{0}=3$ and as $\chi^{2}\left( 1 \right)\approx3.841 \leftrightarrow p=.05$we get

$$3=\exp(-3.841+\log\left( n_{0} \right))/2)$$

$$\leftrightarrow\log\left( n_{0} \right)=2\log\left( 3 \right)+3.841$$

$$\leftrightarrow n_{0}\approx419$$

(In the other direction for $\mathrm{BF}_{0}=\frac{1}{3}$ we get $n_{0}=exp(2\log\left( \frac{1}{3} \right)+3.841)\approx5$)

By loosely citing Dienes (2014) we can use the Bayes factor for the supporting interpretation of only the nonsignificant results. In these cases, $BF>\mathrm{BF}_{0}$ and all nonsignificant effects support the null. On the other hand, all significant effects follow the frequentist interpretation that the alternative is true. As we are dealing with uncertain data or an uncertain world depending on statistical interpretation, this should not happen. We thus deem it necessary to interpret Bayes factors for all effects if they are used to aid interpretation of frequentist analyses.

As demonstrated by Wagenmakers (2007) or the Jeffreys-Lindley paradox, the conclusions of frequentist and Bayesian statistics might be divergent with increasing data. While frequentist favours rejecting the null, Bayesian will favour the null. By combining both theories a region of uncertainty is introduced, where both approaches disagree (assuming for simplicity some equivalency with frequentists not rejecting the null and Bayesians actually favouring the null). The size of this region can be easily calculated by the calculations above. Note that a disagreement typically occurs when Bayesians favour the null while frequentists favour the alternative. This applies to well-known examples against frequentist statistics: When n is large enough, anything will become significant. The opposite direction might theoretically occur for very small sample sizes (less than 5 in the example above).

# References

Bates, D., Kliegl, R., Vasishth, S., & Baayen, H. (2015). Parsimonious Mixed Models. *ArXiv Preprint ArXiv:1506.04967*. http://arxiv.org/abs/1506.04967

Dienes, Z. (2008). *Understanding Psychology as a Science: An Introduction to Scientific and Statistical Inference.* Palgrave Macmillan.

Dienes, Z. (2014). Using Bayes to get the most out of non-significant results. *Frontiers in Psychology*, *5*, 781. https://doi.org/10.3389/fpsyg.2014.00781

Lewis, F., Butler, A., & Gilbert, L. (2011). A unified approach to model selection using the likelihood ratio test. *Methods in Ecology and Evolution*, *2*(2), 155–162. https://doi.org/10.1111/j.2041-210X.2010.00063.x

Lindley, D. V. (1957). A Statistical Paradox. *Biometrika*, *44*, 187–192.

Matuschek, H., Kliegl, R., Vasishth, S., Baayen, H., & Bates, D. (2017). Balancing Type I error and power in linear mixed models. *Journal of Memory and Language*, *94*, 305–315. https://doi.org/10.1016/j.jml.2017.01.001

Wagenmakers, E. J. (2007). A practical solution to the pervasive problems of p values. In *Psychonomic Bulletin and Review* (Vol. 14, Issue 5, pp. 779–804). Psychonomic Society Inc. https://doi.org/10.3758/BF03194105

1. Similarly, a monotonous relationship between the Bayes factors using the calculator associated with Dienes (2008), the t-values, and the p-values emerges if comparable priors are used. [↑](#footnote-ref-1)
